# Electromagnetic stimulation as coadjuvant in the healing of diaphyseal femoral fractures: a randomized controlled trial

**Published:** 2014-06-30

**Authors:** Alfredo Martinez-Rondanelli, Juan Pablo Martinez, María E Moncada, Eliana Manzi, Carlos Rafael Pinedo, Hector Cadavid

**Affiliations:** 1 Universidad del Valle, Cali, Colombia; 2 Fundación Valle del Lili, Cali, Colombia; 3 Instituto Tecnológico Metropolitano, Medellín, Colombia

**Keywords:** Femur fracture, non-union, electromagnetic stimuli, bone healing

## Abstract

**Introduction:**

There is controversy in medical literature regarding the use of electromagnetic fields to promote bone healing.

**Methods:**

After designing and building devices capable of generating an electromagnetic field for this study, their safety was confirmed and the electromagnetic therapy was randomly allocated and compared to placebo in patients with fracture of the femoral diaphysis. Treatment began six weeks after the fracture and it was administered once a day, during 1 h, for eight consecutive weeks. Twenty device were built, 10 of which were placebo-devices. Between June 2008 and October 2009, 64 patients were randomized in two different hospitals and were followed for 24 weeks. The mean age was 30 years (18-59) and 81% were males.

**Results:**

Healing observed at week 12 was 75% vs. 58% (*p* =0.1); at week 18 it was 94% vs. 80% (*p*= 0.15); and at week 24 it was 94% vs. 87% (*p*= 0.43) for the device group and the placebo group, respectively.

**Discussion:**

This study suggests that an electromagnetic field stimulus can promote earlier bone healing compared to placebo in femoral diaphyseal fractures. Faster bone healing translates into sooner weight bearing, which -in turn- permits quicker return to normal daily activities.

## Abstract (es)

**Introducción:**

El uso de estimulación electromagnética como coadyuvante en la consolidación de fracturas es controversial en la literatura médica.

**Métodos:**

Para este estudio, se diseñó y construyó un dispositivo capaz de generar un campo electromagnético. Tras confirmar su seguridad se asignaron pacientes aleatoriamente con fractura diafisaria de fémur a recibir terapia electromagnética o placebo. La estimulación inició a las seis semanas de la fractura, 1 h diaria, por ocho semanas consecutivas. Se construyeron 20 dispositivos, 10 reales y 10 dispositivos-placebo. Entre junio 2008 y octubre 2009, ingresaron 64 pacientes al estudio de dos instituciones y fueron seguidos durante 24 semanas. El promedio de edad de los pacientes fue de 30 años (rango 18-59) y 81% eran de sexo masculino.

**Resultados:**

La consolidación observada para el grupo con el dispositivo y el grupo placebo fue: en la semana 12, 75% vs. 58% (*p*= 0.1); en la semana 18, 94% vs. 80% (*p*= 0.15) y en la semana 24, 94% vs. 87% (*p*= 0.43).

**Discusión:**

Este estudio muestra una tendencia a la consolidación más temprana al estar expuesto a un campo electromagnético frente a placebo. Una consolidación más temprana permite un apoyo precoz y, así, más rápida reincorporación al trabajo y a las actividades cotidianas.

## Introduction

Non-unions and delayed unions are common complications in the treatment of long-bone fractures like the femur 1. In Colombia, up to 25% of femoral fractures do not heal after six months of treatment 2, which raises socio-economic implications to both patients and society 3.

Electromagnetic stimulation, using a device that generates an electromagnetic field, is a non-invasive method to improve fracture union success 4. Stimulation is applied by placing the device around the affected limb during short periods of time for a previously defined number of weeks. This method was empirically used since 1841, but it was not until 1957 when Fukada and Yasuda suggested a relationship between electric stimulation and the formation of bone callus 5 ^,^ 6. More recent studies have demonstrated that electromagnetic stimulation promotes different cellular processes including synthesis of growth factors, collagen fibers, proteoglycans and cytokines.

Previous clinical trials have explored the effects of electromagnetic stimulation in fracture healing with contradictory results 7 ^-^ 15. Methodological limitations and heterogeneity from these studies do not properly showcase the effects of electromagnetic stimulation on bone healing 12. This study specifically evaluates the effects of electromagnetic stimulation for diaphyseal femur fractures using a device developed by the investigators. 

## Materials and Methods

### Participants and study design

The study took place between June 2008 and October 2009 in two medical centers in Cali, Colombia: Fundación Valle del Lili and Hospital Universitario del Valle. The ethics committee of both institutions approved the study. The inclusion criteria were: patients of any sex, age between 18 and 60 years, with a closed fracture in their femoral diaphysis or an open fracture secondary to a low speed bullet; treated with open or closed reduction and intramedullary reamed blocked nail. Patients were excluded if they had a pathological fracture, an open fracture from another etiology, or if they were treated after 10 days from the day of fracture.

**Figure 1.**
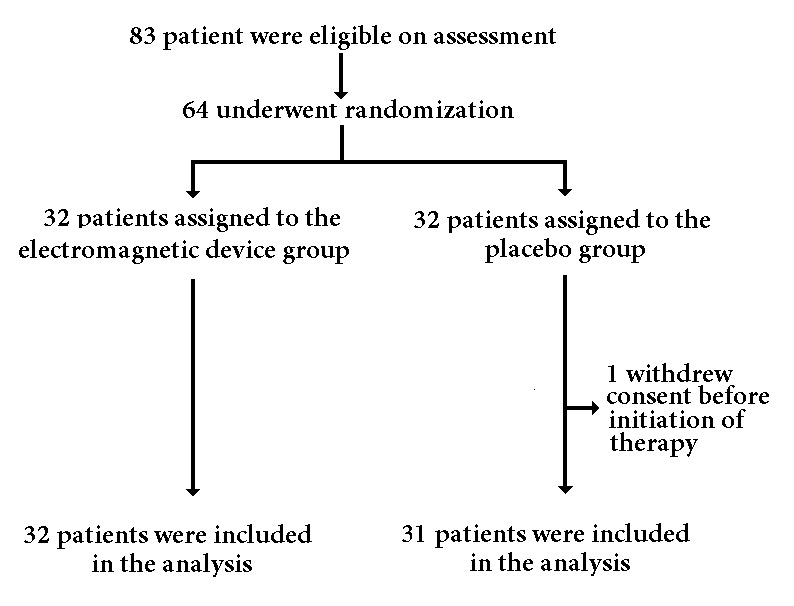
Randomization and treatment of the study subjects.

Eighty three patients fulfilled the eligibility criteria, 64 patients signed the consent form and were included. One patient withdrew consent, leaving a total of 63 participants. This is shown in Figure 1. Randomization was done six weeks after the day of fracture by using random numbers and allocating participants to either one of two groups: group A, assigned to the electromagnetic stimulation device, or group B, which received a placebo device, in a 1:1 ratio. Patients in both groups took the device to their homes where they were instructed on its use during one hour every day for eight weeks. Patients, physicians, and researchers were blinded and could not recognize between real-devices and placebo-devices. A technician installed the device at home and taught the patient and relatives how to properly use the device. Every day, each patient received a call from a health-care worker asking if they had used the device, at what time and for how long, as a way to check and improve adherence to the therapy. Every week, a technician visited the patients, calibrated the device, and checked for how long the therapy was done in the device record.

For the primary outcome measurement (fracture union), a femur X-ray was taken at the 6^th^, 12^th^, 18^th^ and 24^th^ week and it was evaluated by a radiologist, blind to the type of intervention. The radiologist classified the fracture healing in one of three alternatives: non-union, partial union, or complete union. For the secondary outcomes, data was obtained during the visit to the orthopedic surgeon. This information included: comparative length of lower limbs, amount of pain, type of gait, need for crutches, range of motions of the knee, muscle tropism, deformities, infection, alcohol consumption, smoking status, and any other adverse events. Medical visits were in the 6^th^, 12^th^, 18^th^ and 24^th^ week after the fracture.

### Application of the device

**Figure 2.**
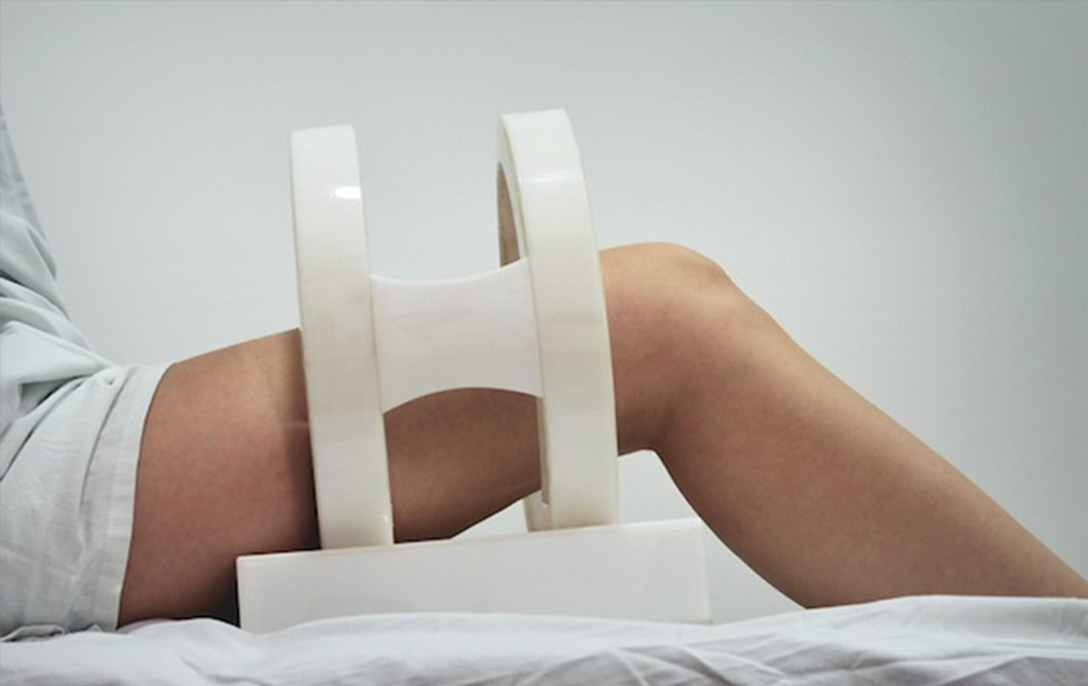
A patient during therapy with the device.

For nine months, 20 devices were designed and built, 10 of which were placebo-devices. Their ability to generate an electromagnetic field was tested; the real devices should induce voltage and current signals in the thigh tissues without any physical contact 16. Each device included a programmable power supply to feed a Helmholtz coil and allowed a digital selection of the magnetic field magnitude to be applied, its frequency, wavelength, and duration. Data on its use was stored in an internal memory. Depending on thigh diameter, a different coil radius could be used: 10, 12.5, or 15 cm. Figure 2 shows the device placed around the thigh of a patient. Every two months, a general technical verification was conducted on each device. Prior to its application on patients, a 3-D computer model of the stimulation coils was constructed by using the ANSYS^®^ software tool. This model was used to test for the electric and magnetic signals produced. The computer data was compared with the values measured. The model was created for low-frequency sinusoidal signals (5-105 Hz) and magnetic fields between 0.5-2.0 mT (modified via programming). Electromagnetic analyses were performed by using the ANSYS^®^ program.

### Statistical analysis

Sample-size calculation was based on a clinically relevant difference in bone union of at least 30% during the 18 weeks of treatment. The defined number of 30 patients per study group provided a power of 80% to detect 50% difference in bone union, with an alpha level of 0.05. Demographic characteristics for patients and outcomes were described according to their group. For quantitative variables, the summary measure used was mean ± standard deviation. Meanwhile, qualitative variables were expressed as percentages. Normality was evaluated with the Shapiro-Wilk test; Student's t-tests were used for comparing means, and both chi-square tests and Fisher's exact tests were used for percentages. Odds ratios were calculated as measures of effect size. *p* values inferior to 0.05 were considered statistically significant. All analyses were performed on an intention-to-treat basis. All statistical analyses were performed with STATA^®^, version 12.0.

## Results

Baseline characteristics such as age, gender, race, etiology, fracture severity and fracture site were similar between groups. The mean age among patients was 30.2 years, the ratio between men and women was 4:1, and most suffered a motor vehicle accident. Active smokers and those who quit smoking in the past six months were grouped together as smokers. For smoking and type of reduction (open vs. closed) no statistically significant difference was noted. All of this is shown in Table 1.

**Table 1.**
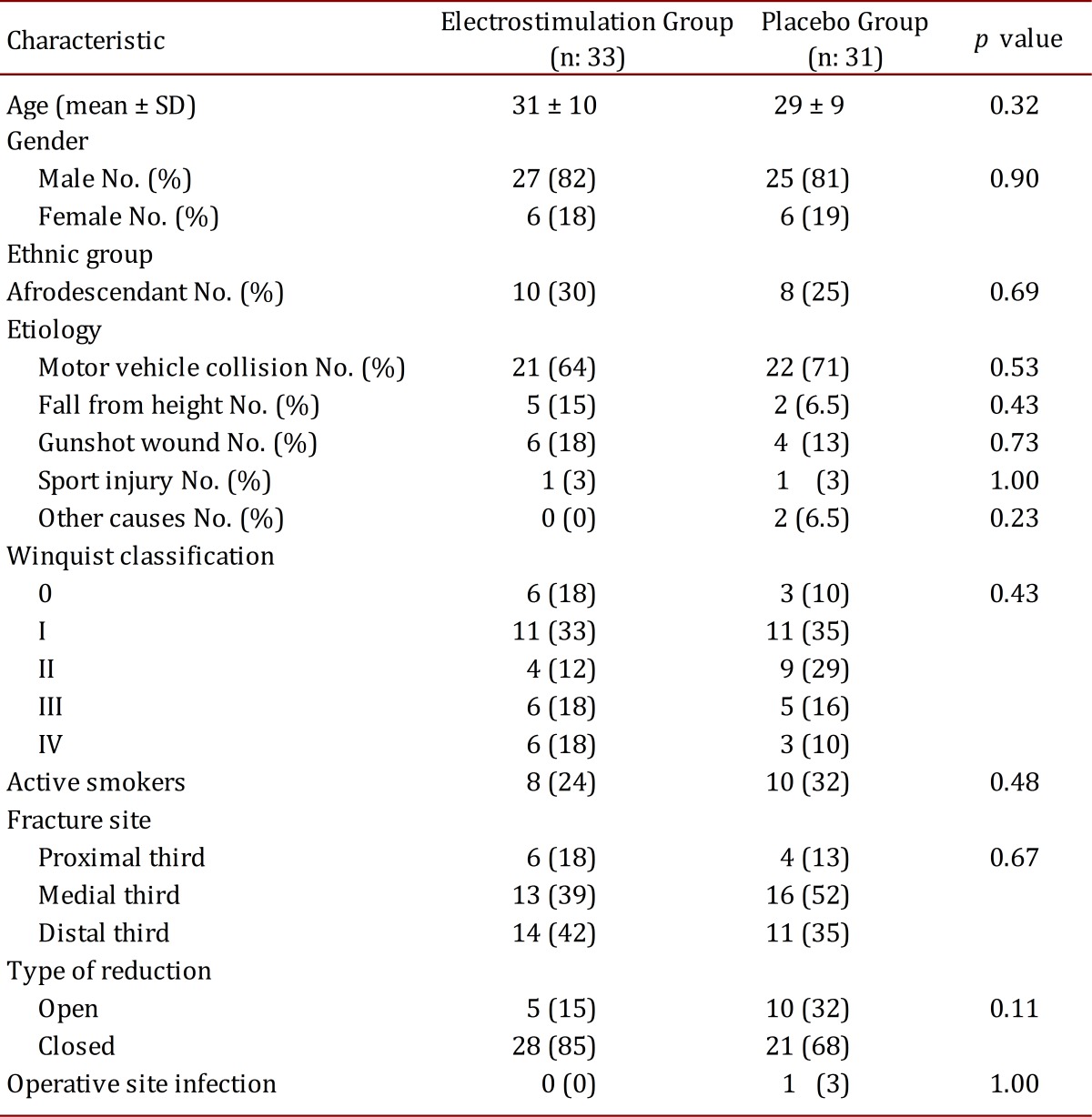
Baseline characteristics of the study patients

**Figure 3.**
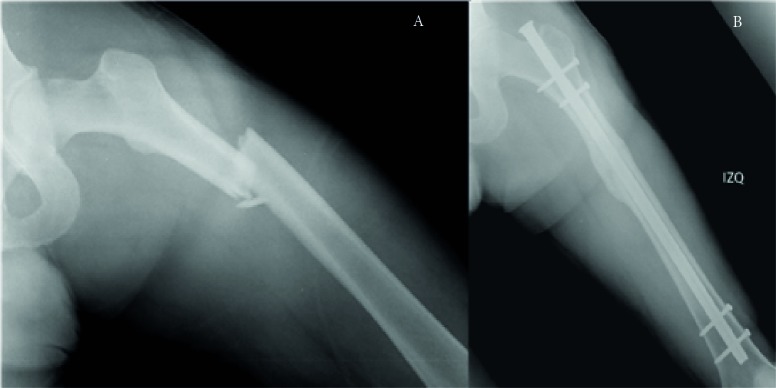
A and B shows the X-rays of one of the participants in the device-group at baseline time and after 24 weeks

In all cases, the fracture was surgically treated within the first 10 days from the initial trauma, but only for 21 patients (33%) was it treated during the first 48 hours. All surgeries were planned as a closed reduction and intramedullary nailing, but 23% needed an open reduction of the fracture, with no difference between groups. In all cases, the nail was reamed and blocked both proximally and distally. Figures 3A and B show a patient with a femoral fracture in the device-group at day 1 and 24 weeks after surgical treatment with this kind of nail. No infections or complications related to surgery or to the use of the devices were apparent. 

Patients whose X-rays showed union (complete or partial), were grouped together and compared against patients with non-union X-rays. As shown in Table 2, healing observed at week 12 was 75% vs. 58% (*p*= 0.1), at week 18: 94% vs. 80% (*p*= 0.15), and at week 24: 94% vs. 87% (*p*= 0.43) for device group and placebo group, respectively. This shows a non-statistically significant difference in the time in which fractures progress to union. The risk of non-union was lower for the electromagnetic stimulation group at the three measure points: week 12 (relative risk for non-union, 0.60; 95% CI, 0.29 to 1.24), week 18 (RR: 0.32; 95% CI, 0.07 to 1.48) and week 24 (RR: 0.70; 95% CI, 0.17 to 2.88). 

**Table 2.**
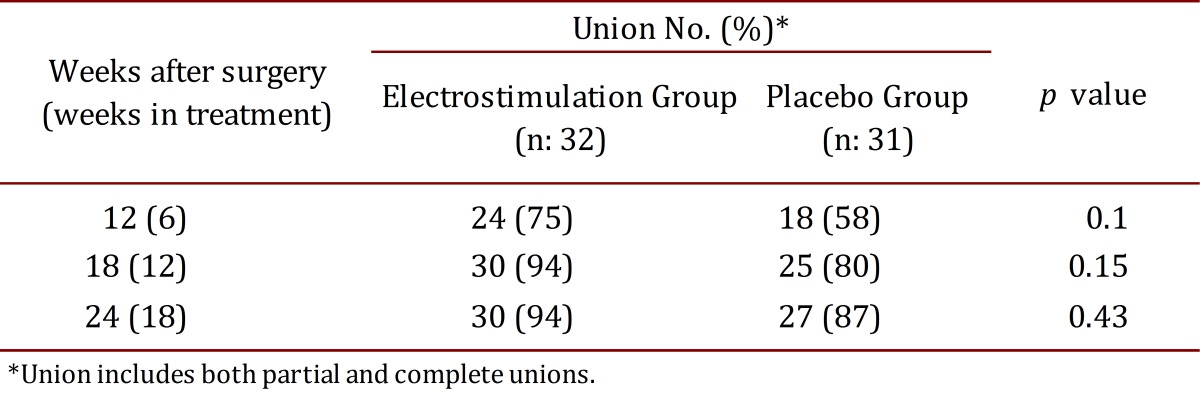
Fracture union according to study device

The following complications unrelated to the treatment group were observed: 11 patients (17%) had limb shortening, all of them between 1.0 and 2.0 cm. 12 patients (19%) had a rotational deformity greater or equal to 15° (most were externally rotated). All patients, except two, had a knee flexion of at least 90°. No differences in adherence and total time of treatment were noted. 

## Discussion

Diaphyseal femur fractures have a union rate between 90-100% depending on the series 3 ^,^ 17; Nevertheless, in the clinical practice non-union continues occurring frequently. Currently, it can be higher than 10% given the introduction of damage control techniques for polytraumatized patients and the development of improved limb salvage methods. In Colombia, reports are available where femoral union is only achieved in 85% of the patients 2.

Several factors may affect fracture healing. Non-modifiable factors like age, type of fracture, bone quality, or co-morbidities; and modifiable factors like surgical technique, nutritional status, active smoking, type of physical therapy, time to weight bearing, quality of fracture reduction and fixation. Interventions at this level may improve bone healing, but there are still patients whose fractures do not heal. Randomization allows having these variables equally distributed among patients. The rehabilitation program was similar for both treatment groups. 

The electromagnetic device designed for this study was built for the purpose of testing a new coadjuvant to heal fractures in our patients. It represents a non-invasive, cheap, safe and easily administered method; Though, electromagnetic fields have been studied previously with no conclusive results about their effectivity 4 ^-^ 15. 

In this study, bone healing was achieved faster when patients were exposed to an electromagnetic field compared to the placebo group. At week 12, unions were achieved in 75% vs. 58% (*p*= 0.1), and at week 18, unions were achieved in 94% vs. 80% (*p*= 0.15). This shows a trend of more patients having their fracture healed in less time when using the electromagnetic field as coadjuvant. Nevertheless, this was not statistically significant difference. At the end, after 18 weeks of electromagnetic therapy or 24 weeks since the fracture day, no differences were identified between both groups, 94% vs. 87% (*p*= 0.43). The difference in bone healing time between groups decreases as weeks pass until they become virtually the same at week 24. 

This trend in bone healing speed has important implications. Faster healing translates into earlier weight bearing, improved function and independence, which then can facilitate sooner return to work and daily activities with the social, psychological, and economical repercussions this brings. Costs of disability, temporary cessation of work, babysitting or nursing, may decrease if the patient recovers earlier. Therefore, every effort must be made to obtain quicker bone healing in patients, especially in fractures that like the femur can take between four and six months to heal.

Probably a larger sample size could have given the sufficient power to show a statistically significant difference in bone union at week 12 and week 18. Even so, the data from this study can be further used in systematic reviews and the pooled information from all trials would certainly increase sample size and would shorten the distance to find a statistically significant difference, if it indeed exists.

## Conclusion

Electromagnetic stimulation is a safe therapy that may be used as coadjuvant for bone consolidation. It might accelerate the process of bone healing for long-bone fractures. More studies are needed to confirm this effect. 
